# PA0833 Is an OmpA C-Like Protein That Confers Protection Against *Pseudomonas aeruginosa* Infection

**DOI:** 10.3389/fmicb.2018.01062

**Published:** 2018-05-23

**Authors:** Feng Yang, Jiang Gu, Jintao Zou, Langhuan Lei, Haiming Jing, Jin Zhang, Hao Zeng, Quanming Zou, Fenglin Lv, Jinyong Zhang

**Affiliations:** ^1^College of Bioengineering, Chongqing University, Chongqing, China; ^2^National Engineering Research Center of Immunological Products, Department of Microbiology and Biochemical Pharmacy, College of Pharmacy, Third Military Medical University, Chongqing, China; ^3^Department of Critical Care Medicine, Children’s Hospital of Chongqing Medical University, Chongqing, China

**Keywords:** *Pseudomonas aeruginosa*, PA0833, OmpA, pneumonia, sepsis

## Abstract

*Pseudomonas aeruginosa* is a formidable pathogen that causes infections with high mortality rates. Because of its ability to form biofilms and rapidly acquire resistance to many first-line antibiotics, *P. aeruginosa*-related infections are typically difficult to cure by traditional antibiotic treatment regimes. Thus, new strategies to prevent and treat such infections are urgently required. PA0833 is a newly identified protective antigen of *P. aeruginosa* that was identified in a screen using a reverse vaccine strategy in our laboratory. In this study, we further confirmed its protective efficacy in murine sepsis and pneumonia models. Immunization with PA0833 induced strong immune responses and resulted in reduced bacterial loads; decreased pathology, inflammatory cytokine expression and inflammatory cell infiltration; and improved survival. Furthermore, PA0833 was identified as an OmpA C-like protein by bioinformatics analysis and biochemical characterization and shown to contribute to bacterial environmental stress resistance and virulence. These results demonstrate that PA0833 is an OmpA C-like protein that induces a protective immune response in mice, indicating that PA0833 is a promising antigen for vaccine development.

## Introduction

*Pseudomonas aeruginosa* (*P. aeruginosa*) is a leading cause of hospital-acquired infections (Kalil et al., 2016), especially in patients with cystic fibrosis or compromised host defense mechanisms (Nathwani et al., 2014; Kizny Gordon et al., 2017). *P. aeruginosa* can infect any part of the body, and serious *P. aeruginosa* infections, such as infections of the blood, pneumonia, and infections following surgery, are typically complicated and can be life-threatening (Gellatly and Hancock, 2013). These infections are generally treated with antibiotics (Curran et al., 2017). Unfortunately, because of increasing antibiotic resistance and the ability of *P. aeruginosa* to form biofilms, such infections are becoming more difficult to treat, imposing a high and increasing burden on health care resources (Melsen et al., 2013; Ramirez-Estrada et al., 2016).

Vaccination strategies to prevent *P. aeruginosa* infection have attracted a great deal of attention. In the past two decades, a number of different vaccines and several monoclonal antibodies have been developed for active and passive vaccination against *P. aeruginosa* infection (Pier, 2005; Priebe and Goldberg, 2014; Vincent, 2014; Grimwood et al., 2015; Le Moigne et al., 2016). The antigens used in these studies include lipopolysaccharides (Pier, 2007), surface polysaccharides (Pier, 2000), outer membrane proteins (Gao et al., 2017; Rello et al., 2017), secreted proteins (Hamaoka et al., 2017; Yang et al., 2017), flagella (Hassan et al., 2017), and pili (Ohama et al., 2006; Banadkoki et al., 2016). Several vaccine candidates even entered phase II/III clinical trials (Baumann et al., 2004; Pier, 2005; Sharma et al., 2011; Le Moigne et al., 2016; Rello et al., 2017). However, these efforts have not resulted in a marketable product to date, which can be attributed to two primary reasons. First, *P. aeruginosa* utilizes multiple virulence factors and pathways to establish a successful infection, and immunization with a single antigen is able to provide only partial protection (Gellatly and Hancock, 2013; Curran et al., 2017). Second, it is unclear what type of immune response should be induced by an effective *P. aeruginosa* vaccine to clear an infection (Worgall, 2012). Thus, more effort should be focused on studying the pathogenic mechanisms of *P. aeruginosa* and identifying new candidate antigens.

Our lab has been working on developing a *P. aeruginosa* vaccine since 2011, and we have identified several novel candidate antigens, such as PA0833, PA5505, PA4110, and PA0807. PA0833 is a hypothetical, uncharacterized protein that is 237 amino acids in length and has an unclear structure and function. Only one study (Choi et al., 2011) has demonstrated that PA0833 is a component of outer membrane vesicles secreted by *P. aeruginosa*. In the present study, we observed that PA0833 is a promising candidate antigen for vaccine development. It exhibited strong immunogenicity in mice and was able to protect mice against a *P. aeruginosa* challenge in both sepsis and acute lung infection models. Further bioinformatics prediction and biochemical studies indicated that PA0833 has characteristics of the C-terminal domain of OmpA family proteins and that it may be an OmpA family protein. Moreover, this protein may be involved in the pathogenesis of the bacterium by affecting the synthesis of alginate. These results will be helpful for understanding the biological function of PA0833 and enabling future studies of the pathogenic mechanism of this protein.

## Materials and Methods

### Ethics Statement

All animal care and use protocols in this study were performed in accordance with the Regulations for the Administration of Affairs Concerning Experimental Animals approved by the State Council of the People’s Republic of China. All animal experiments in this study were approved by the Animal Ethical and Experimental Committee of the Third Military Medical University (Chongqing, Permit No. 2011-04) in accordance with their rules and regulations. All surgery was performed under sodium pentobarbital anesthesia, and all efforts were made to minimize suffering.

### Bacterial Strains and Culture Methods

The *P. aeruginosa* standard strain PAO1 was purchased from ATCC (Manassas, VA, United States). Bacterial strains were cultured in Luria Bertani (LB) broth (Difco Laboratories, United States), washed and diluted with sterile phosphate buffer solution (PBS) to an appropriate cell concentration determined spectrophotometrically at 600 nm (OD_600_).

### Animals

The 8–12-week-old female BALB/c mice used in this study were purchased from Beijing HFK Bioscience Limited Company (Beijing, People’s Republic of China). Mice were matched for age and sex and were housed under specific pathogen-free (SPF) conditions. All mice were randomly assigned to treatment groups using a randomization tool implemented in MS Excel. Female New Zealand white rabbits (2.00 ± 0.20 kg) were provided by TengXin Company (Chongqing, China).

### Bioinformatics Prediction of PA0833

SignalP 4.0 (Petersen et al., 2011), TMHMM 2.0 (Chen et al., 2003), CFSSP, Phyre2 (Kelley et al., 2015), and TBBprep (Natt et al., 2004) were used to predict the possible signal peptide, trans-membrane domain, secondary structure and homologous proteins of PA0833, respectively. The structure of PA0833 was modeled by the automated protein homology-modeling server SWISS-MODEL (Arnold et al., 2006). Sequence alignment was performed using the online software ClusterW at http://www.ebi.ac.uk/Tools/msa/clustalw2/.

### Cloning and Expression of Recombinant PA0833

Two constructs were made to produce truncated PA0833 proteins comprising residues 26–237 and 86–237. In brief, the coding sequences of the two constructs were amplified by PCR from *P. aeruginosa* strain PAO1 genomic DNA and cloned into the pGEX6p-2 vector (Novagen, Italy) via the *Bam*H1/*Xho*I restriction sites. The inserts were sequenced and determined to be in complete agreement with the expected sequences. The resulting proteins harbor an N-terminal glutathione S-transferases (GST) tag to facilitate the purification of the proteins. *Escherichia coli* strain BL21 (DE3) competent cells (Tiangen Biotech, China) were transformed with the recombinant plasmids, and isopropyl-β-d-thiogalactopyranoside (IPTG) was then added to a final concentration of 0.05 mM to induce the expression of recombinant protein at 16°C overnight.

### Purification and Characterization of Recombinant PA0833

GST-tagged proteins were harvested from cleared lysates with glutathione-Sepharose, and the GST tag was cleaved using PreScission Protease (GE Healthcare, United States). Next, the recombinant proteins were purified by hydrophobic chromatography using a 5 ml phenyl HP column. Finally, the proteins were desalted with PBS and loaded onto a 5 ml Q HP column to remove endotoxins. The purity and concentration of the proteins was determined by sodium dodecyl sulfate-polyacrylamide gel electrophoresis (SDS–PAGE) and the bicinchoninic acid (BCA) Protein Assay Kit (Thermo scientific, United States), respectively. The endotoxin content after the proteins were purified was detected using the kinetic turbidimetric tachypleus amoebocyte lysate assay (Houshiji cod Inc., Xiamen, China), and the endotoxin of the two recombinant proteins were at acceptable levels (<2.5 pg/μg).

The oligomeric states of PA0833_86-237_ were analyzed by using a Superdex^TM^ 200 10/300GL column as described previously (Zhang et al., 2013). Gel Filtration Calibration Kits (GE Healthcare, United States) was used to generate the calibration curve, 200 μl purified PA0833_86-237_ was loaded onto the column, and the elution volume of the corresponding peak was used to calculate the molecular weight, thereby determining the oligomeric state of the protein.

PA0833_86-237_ were further analyzed by chemical cross-linking analysis following the protocol described by Fadouloglou (Fadouloglou et al., 2008). In brief, PA0833_86-237_ or bovine serum albumin (BSA) was diluted to 1 mg/ml and incubated with glutaraldehyde at 37°C for 30 min. The final concentration of glutaraldehyde in each reaction was 0.01, 0.05, 0.1, 0.2, 0.3, 0.4, and 0.5%. The cross-linking reaction was then terminated by adding loading buffer containing SDS and glycine. The protein samples were then analyzed by SDS–PAGE.

### Peptidoglycan Binding Assay

The peptidoglycan binding assay was performed as described previously with minimal modifications (Wang et al., 2016). First, 50, 100, 150 or 200 μg of commercial insoluble peptidoglycan (InvivoGen, United States) was incubated with PA0833_86-237_ (0.5 mg/ml) or BSA (0.5 mg/ml) in 10 mM sodium phosphate buffer with 50 mM NaCl and pH 7.5 for 1 h at room temperature. After the mixtures had been centrifuged for 20 min 12,000 g, the supernatants were collected, and the precipitates were washed three times with 10 mM sodium phosphate buffer (with 500 mM NaCl, pH 7.5). Finally, the precipitates were resuspended in sodium phosphate buffer. The supernatant and precipitate fractions were analyzed by western blotting using rabbit anti-PA0833_86-237_ or anti-BSA polyclonal antibodies as the primary antibodies. The anti-PA0833_86-237_ polyclonal antibodies (pcAb) were generated in rabbits based on a previously published method (Zhang et al., 2015).

### Immunization of Mice

Purified PA0833_26-237_ in Histidine (His) buffer (10 mM His, 150 mM NaCl, pH 6.0) was emulsified 1:1 (v/v) in Al(OH)_3_ (Pierce, United States). Mice were intramuscularly injected with 200 μl of the emulsion containing 30 μg protein, His buffer plus adjuvant, or His buffer alone as the control on days 0, 14, and 21, and the mice were infected on day 35.

### ELISA

On day 28 after primary immunization, mice were exsanguinated, and serum samples were collected for an enzyme-linked immunosorbent assay (ELISA). Microtiter plate wells (Corning Incorporated, United States) were coated with PA0833_26-237_ (200 ng per well) in 0.05 M carbonate buffer (pH 9.5) overnight at 4°C. Diluted serum samples were used as the primary antibodies, and the secondary antibodies were horseradish peroxidase (HRP)-conjugated goat anti-mouse IgG, anti-mouse IgG1, anti-mouse IgG2a or anti-mouse IgG2b (Sigma). The optical density at 450 nm was measured, and the titers were defined as the highest dilution that yielded an absorbance value of more than twice the value of the pre-immune serum.

### *P. aeruginosa* Sepsis Mouse Model

To measure the survival rates of mice in the *P. aeruginosa* sepsis model, immunized BALB/c mice were intravenously infected with PAO1 [7.0 × 10^7^ colony-forming units (CFUs)] on day 35 and monitored for survival for 14 days after infection. For bacterial burdens in the sepsis model, a sub-lethal dose (3.5 × 10^7^ CFUs) of PAO1 was intravenously administered to each mouse. Livers and spleens from all immunized mice were removed, weighed, and homogenized in 1 ml of PBS 1 or 3 days after infection. The peripheral blood was collected in heparin anticoagulant tubes. All samples were then plated on LB plates at a 10-fold serial dilution and cultured at 37°C for 20 h. The number of CFUs per gram of tissue (CFUs/g) was calculated from each plate.

### *P. aeruginosa* Pneumonia Mouse Model

In the acute pneumonia model, mice in each group were anesthetized with pentobarbital sodium followed by intratracheal injection with a lethal dose of PAO1 (1.0 × 10^7^ CFUs) to measure the survival rates. The number of deaths in each group was recorded every 12 h over a 7-day observation period post challenge. For bacterial burdens, histopathology, inflammatory cells and cytokine analyses, mice were infected by intratracheal injection with 5.0 × 10^6^ CFUs of PAO1. Next, the lung tissues were collected, weighed, and homogenized in 1 ml of sterilized PBS buffer 24 h after infection to determine CFUs.

### Histological Analysis

Lung tissues were collected from the pneumonia model mice 24 h post-infection, inflated, and fixed in 10% neutral buffered formalin. All lung samples were embedded in paraffin, sectioned, and stained with hematoxylin and eosin (HE). The sections were then viewed at 200 × magnifications by a single pathologist who was blinded to the study groups. Each lung section was given a score of 0–4 (no abnormality to most severe) according to established criteria based on hyperemia, edema, hemorrhage, and neutrophil infiltration (Zuo et al., 2013).

### Evaluation of Inflammation

To quantify the neutrophils’ infiltration of lungs in the pneumonia model, cells in bronchoalveolar lavage fluid (BALF) from mice 24 h post challenge were collected and stained using the following antibodies: PE/Cy7 anti-mouse CD45 and APC/cy7 anti-mouse Ly-6G (Biolegend Inc., United States). Samples were then analyzed using BD FACSArray software on a BD FACS Array flow cytometer (BD Biosciences).

To quantify proinflammatory responses, cytokines such as TNF-α, IL-1β, and IL-6 in BALF were collected 24 h after infection. The concentrations of proinflammatory cytokines were determined using a Mouse Quantikine ELISA kit for TNF-α, IL-1β or IL-6 (R&D Systems, United States) according to the manufacturer’s instructions.

### Construction of PAO1 Isogenic Mutants

The PA0833 knockout strain (PAO1/ΔPA0833) was constructed using the suicide vector pCVD442 (Liu et al., 2009). Briefly, the primers were designed to amplify the gentamicin resistance (GmR) gene from the plasmid pJQ200SK (primers P1 and P2 in **Table 1**, PCR conditions in Supplementary Table S1) and amplify the 5′- and 3′-regions of PA0833, including flanking sequences (primers P3/P4 and P5/P6 in **Table 1**, PCR conditions in Supplementary Table S2). The three fragments were joined (primers P3 and P6 in **Table 1**, PCR conditions in Supplementary Table S3) and cloned into the suicide plasmid pCVD442 to yield the plasmid pCVD442-ΔPA0833::GmR, which was then transformed into *E. coli* β2155 (Sangon Biotech, China), and then positive clones were selected on LB agar containing ampicillin (100 μg/ml), gentamicin (25 μg/ml) and diaminopimelic acid (DAP, 0.5 mM). Next, conjugation between the recipient PAO1 and the donor β2155/pCVD442-ΔPA0833::GmR was performed to transfer the recombinant suicide plasmid pCVD442-ΔPA0833::GmR from β2155 to PAO1. Gentamicin- and sucrose-resistant colonies were selected and screened by PCR using primers P7 and P8, as shown in **Table 1**. These primers yielded fragments of 2490 and 2695 bp for the intact and mutated GmR gene, respectively. To further characterize the GmR gene in one of the selected mutant strains, primers P9 and P10 (shown in **Table 1**) were used for its amplification and confirmation by DNA sequencing (Sangon Biotech, China).

**Table 1:** Primers used in this study.

Name	Sequences (5′ to 3′)
P1	CGAGGAGTAGTTTTCCCATGTTCACCT
	CCCGTAGAAATGCCTCGACTTC
P2	GCCGCGATCAGTACTGCGGCTGTTGAG
	ACAATTTACCGAACAAC
P3	ATATCTAGACCGTGATCGGAGT
	CTTGCATGTCGACATC
P4	ACGGGAGGTGAACATGGGAAA ACTACTCCTCG
P5	CAGCCGCAGTACTGATCGCGGC
P6	ATATCTAGAGCTGGATGAACCT
	GGTCCAGCGCAC
P7	CTGCTGCTCGGCTTCAACGA TTCGTCGAAC
P8	GATCCAGCCTTCGGCGATCA GGCTCATGATC
P9	CAACATCAGCCGGACTCCGATTAC
P10	GTAATCGGAGTCCGGCTGATGTTG
P11	CCGGAATTCATGTTCACCTCCCGTTG
P12	CCCAAGCTTTCAGTACTGCGGCTGGGT


To construct PAO1/ΔPA0833 strain complemented with PA0833 (PAO1/CPA0833), plasmid pDN18-PA0833 was constructed by cloning PA0833 gene into the EcoRI/ HindIII site of the broad-host-range vector pDN18 (Bai et al., 2007). This PA0833 gene was amplified by PCR using primers P11 and P12 (**Table 1**) from the chromosomal DNA of PAO1. Resulting plasmid with the PA0833 gene was electroporated into the PAO1/ΔPA0833 strain to generate the strain PAO1/CPA0833. The expression of PA0833 was detected by indirect immunofluorescence, which was carried out based on a method established by us previously (Zhang et al., 2015).

### Analysis of PAO1 Mutant Resistance to Environmental Stress

The growth rate of wild-type PAO1 (PAO1/WT), PAO1/ΔPA0833 and PAO1/CPA0833 was recorded prior to the initiation of experiments. For the acid survival analysis (Wang et al., 2016), bacteria in the exponential growth phase were harvested and adjusted to 3.0 × 10^7^ CFUs/ml in PBS. Next, 1/10th of the bacterial suspension was mixed with LB containing 60 mM acetic acid (pH 4.0) and incubated for 150 min at 37°C, with samples taken bacterial enumeration every 30 min. The relative growth index is the ratio of the number of bacteria after growing in acidic conditions relative to their starting point.

For the SDS resistance assay (Wang et al., 2016), bacteria in the exponential growth phase were harvested and adjusted to 1.0 × 10^9^ CFUs/ml in fresh LB. Next, 1/100th of the bacterial suspension was inoculated in LB containing 9% SDS and incubated for 8 h at 37°C. Samples were taken for determining the absorbance at 600 nm every hour.

### Analysis of the Expression of PA0833 in the GEO Profiles Database

The GEO database stores gene expression profiles derived from curated GEO DataSets. Each profile is presented as a chart that displays the expression level of one gene across all samples within a DataSet. To gain insight into differential expression patterns of PA0833 across different experimental conditions, the PA0833 gene was analyzed using the GEO profiles database^1^.

### Statistical Analysis

Data are presented as the means ± standard deviation (SD) or the means ± standard error of the mean (SEM). Scoring experiments were performed in a blind manner. Survival data were analyzed using Kaplan-Meier survival curves. To calculate *P*-values, non-parametric Mann–Whitney tests, log-rank tests, Student’s *t*-tests, or one-way ANOVA with Bonferroni correction were used depending on sample distribution and variation as described in the figure legends. SPSS 13.0 (SPSS Inc., United States) and GraphPad Prism 6.0 (GraphPad Software, Inc., United States) were used to perform statistical analyses. Significance was accepted at *P* < 0.05.

## Results

### PA0833 Stimulates Protective Immunity in a *P. aeruginosa* Sepsis Model

**FIGURE 1:**
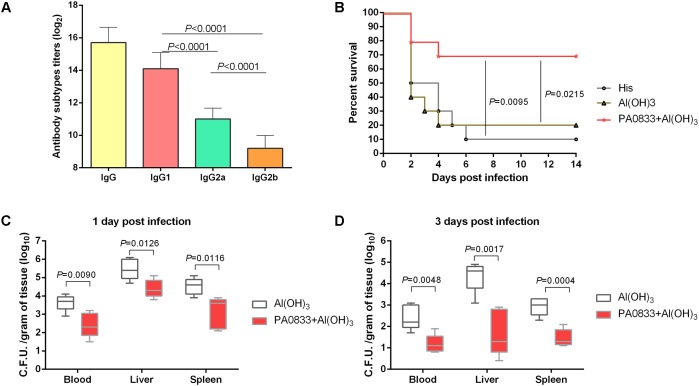
PA0833 stimulates protective immunity in a *P. aeruginosa* sepsis model. **(A)** Comparison of total IgG and IgG subgroups (IgG1, IgG2a, and IgG2b) in the mice (*n* = 5) immunized with PA0833. Serum was obtained at 7 days after the final immunization, and the levels of total IgG and IgG subgroups were expressed as the means of log_2_ titers. Multiple comparisons among different groups were analyzed using one-way ANOVA. Data are shown as the means ± SD. **(B)** BALB/c mice (*n* = 10) were immunized with PA0833 plus an Al(OH)_3_ adjuvant and challenged with PAO1 at 7.0 × 10^7^ CFUs/mouse by intravenous injection. The survival rate was monitored for 14 days. The *P*-values were calculated using the Mantel–Cox log-rank test. **(C,D)** Efficacy of immunization with PA0833 on the spread of *P. aeruginosa*. The number of viable bacteria in the blood, liver, and spleen of mice (*n* = 10) at 1 and 3 days post-infection are shown. Data are presented in box and whisker plots, and the medians are shown. Differences were compared to determine their significance using Student’s *t*-test.

We first tested the immunogenicity of PA0833 in mice. After the last booster immunization, the levels of total IgG and IgG subgroups (IgG1, IgG2a, and IgG2b) in the mice immunized with PA0833 were determined by ELISA. As shown in **Figure 1A**, the geometric mean titer of total IgG was approximately 2^16^, which confirmed that PA0833 is a powerful immunogen. Meanwhile, the level of antigen-specific IgG1 was approximately 32 times higher than that of IgG2a (**Figure 1A**). Since IgG1 and IgG2a are markers for Th2 and Th1 responses, respectively, this result suggested that a Th2-biased response was induced upon immunization with PA0833. We next tested the protective efficacy of PA0833 in a *P. aeruginosa* sepsis model. Two weeks after the last immunization, mice were challenged with a lethal dose of PAO1. As shown in **Figure 1B**, at the end of the observation period, 7 out of 10 mice in the PA0833 formulated with Al(OH)_3_ group survived the PAO1 challenge, which was significantly higher than that in the His buffer control group (*P* = 0.0095) and adjuvant control group (*P* = 0.0215), indicating that PA0833 stimulated protective immunity in mice.

Next, the PA0833-immunized mice were challenged with a sub-lethal dose of *P. aeruginosa* to investigate the bacterial burden in the organs. The results showed that the bacterial burden in the blood, livers, and spleens were much lower in the PA0833 group 1 day post-infection mice than in the Al(OH)_3_ control group (*P*_blood_ = 0.0090, *P*_liver_ = 0.0126, and *P*_spleen_ = 0.0116, **Figure 1C**). Furthermore, the reduction of *P. aeruginosa* was enhanced in the PA0833 group 3 days post-infection compared to that in the Al(OH)_3_ group (*P*_blood_ = 0.0048, *P*_liver_ = 0.0017, and *P*_spleen_ = 0.0004, **Figure 1D**). These results showed that immunization with PA0833 protected mice against *P. aeruginosa* infection by reducing the ability of the bacteria to colonize and directly attack organs, improving the survival of the mice.

### PA0833 Vaccination Protects Mice From Pneumonia by Reducing Local Bacterial Burden and Inflammation

**FIGURE 2:**
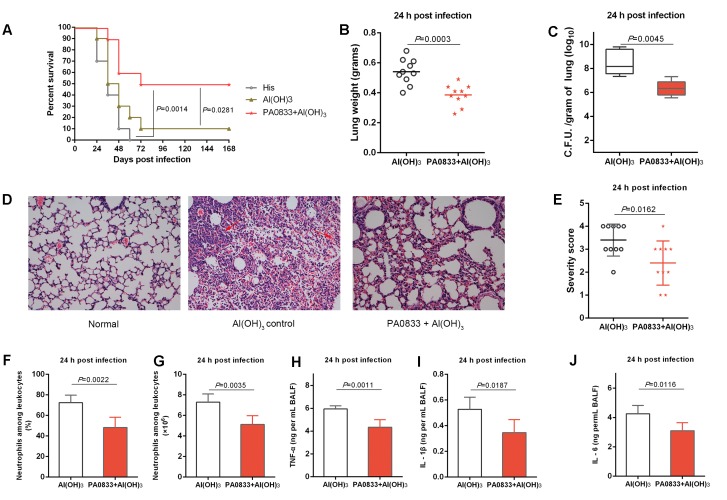
Protective efficacy of PA0833 in a murine *P. aeruginosa* pneumonia model. **(A)** BALB/c mice (*n* = 10) were immunized with PA0833 plus an Al(OH)_3_ adjuvant and challenged with PAO1 at 1.0 × 10^7^ CFUs/mouse by intratracheal injection. The survival rate was monitored for 1 week. **(B–J)** The immunized mice and control mice were infected intratracheally with 5.0 × 10^6^ CFUs/mouse of PAO1. **(B)** Lung weight in infected mice (*n* = 10) immunized with PA0833 plus an Al(OH)_3_ adjuvant. The data are presented as scatter plots. **(C)** The number of viable bacteria in the lungs of mice (*n* = 10) at 24 h post-infection are shown. Data are presented in box and whisker plots, and the medians are shown. **(D)** Hematoxylin-eosin staining of lungs from immunized mice and control mice 24 h after infection. Representative histopathological sections from 10 mice per group are shown (magnification = 200 × ). The arrows point to hemorrhage and neutrophil infiltration in lungs. **(E)** Semi-quantification of lung inflammation in infected mice. Severity scores of lungs (*n* = 10) from immunized mice and control mice 24 h post-infection are shown. The data are presented as scatter plots. **(F,G)** Evaluation of neutrophil infiltration in infected mice (*n* = 10). The bar represents the percentage **(F)** and the number **(G)** of neutrophils in the BALF of immunized mice at 24 h post challenge. **(H–J)** Quantitative detection of proinflammatory cytokines TNF-α, IL-1β and IL-6 in infected mice (*n* = 10). The data **(F–J)** are shown as the means ± SD. The *P*-value **(A)** was calculated using the Mantel–Cox log-rank test. The differences **(B,C,E–J)** were compared to determine their statistical significance using Student’s *t*-test.

PA0833 also showed protective efficacy in a lethal *P. aeruginosa* pneumonia model. Mice were immunized with 30 μg of PA0833 at day 0, 14, and 21 and then intratracheally infected with a lethal dose of PAO1. Similar to the sepsis model, mice exhibited survival rates after being immunized with PA0833 (50%, **Figure 2A**) that were significantly higher than those in the His buffer control group (*P* = 0.0014) and the Al(OH)_3_ adjuvant control group (*P* = 0.0281, **Figure 2A**).

Next, the PA0833-immunized mice were challenged with a sub-lethal dose of *P. aeruginosa* to investigate the mechanism of PA0833-induced protection. First, the lungs from immunized and control mice were harvested 24 h post-infection. Mice that were immunized with PA0833 exhibited decreased pulmonary edema post-infection, as measured by lung weight, compared to the Al(OH)_3_ immunized mice (*P* = 0.0003, **Figure 2B**). In addition, the PA0833 vaccinated group showed significantly lower bacterial loads than the Al(OH)_3_ group 24 h post-infection (*P* = 0.0045, **Figure 2C**).

Histological analysis showed that the lungs from the mice in the PA0833 immunized groups exhibited reduced alveolar disruption, vascular leakage and deposition of bacterial microcolonies in the alveoli after infection compared with the Al(OH)_3_ group (**Figure 2D**). In addition, typical pathological changes were observed in mice immunized with Al(OH)_3_ (**Figures 2D,E**). However, mice immunized with PA0833 exhibited reduced inflammatory cell infiltration, bleeding, and tissue damage compared to the Al(OH)_3_ group (**Figures 2D,E**).

Markers of inflammation, including neutrophil infiltration and proinflammatory cytokine production in the BALF, such as TNF-α, IL-1β and IL-6, were determined 24 h post-infection. Consistent with the lung histopathology results described above, neutrophil number and percentage relative to other leukocytes were significantly reduced in the BALF of mice immunized with PA0833 (**Figures 2F,G**). Similar results were observed for proinflammatory cytokine secretion (**Figure 2H–J**). Taken together, these results confirmed the protective efficacy of PA0833 vaccination, which was attributed to reduced pulmonary edema, bacterial burden, pathology and proinflammatory cytokine production.

### Bioinformatics Prediction Indicates PA0833 Is an OmpA C-Like Protein

Our results clearly showed that PA0833 conferred protection against *P. aeruginosa* infection in animal models, but the biochemical and structural properties of this protein have not been characterized to date. We systematically predicted the bioinformatics features of this protein based on its sequence. According to our results, the C-terminus of PA0833 is similar to that of OmpA family proteins. First, the subcellular localization of PA0833 is predicted to be similar to that of OmpA family proteins. PA0833 consists of two domains, the N-terminal domain contains a signal peptide (amino acids 1 to 25) and two trans-membrane helixes (Supplementary Figure S1A), which may anchor the protein to the membrane of the bacteria. Whereas the C-terminal domain is located in the periplasm. Second, the predicted overall structure of the C-terminal domain of PA0833 (PA0833_86-237_) modeled by SWISS-MODEL contains 3 α-helixes and 3 β-sheets, which share the same folding pattern as the crystal structure of the C-terminal domain of OmpA from *Acinetobacter baumannii* (Park et al., 2012) (Supplementary Figure S1B). Third, when the sequence of the C-terminal region of PA0833 was aligned with homologous proteins, the similarity and identity was approximately 50 and 20%, respectively (Supplementary Figure S1C). Finally, the structure of the C-terminal domain of OmpA from *A. baumannii* revealed that two residues (Asp271 and Arg286) were involved in binding diaminopimelate (DAP), a unique bacterial amino acid present in peptidoglycan, and these residues were completely conserved in PA0833 (Supplementary Figure S1C). These results strongly indicate that PA0833 is an OmpA C-like protein.

### PA0833 Exists as a Dimer in Solution and Binds to Peptidoglycan *in Vitro*

**FIGURE 3:**
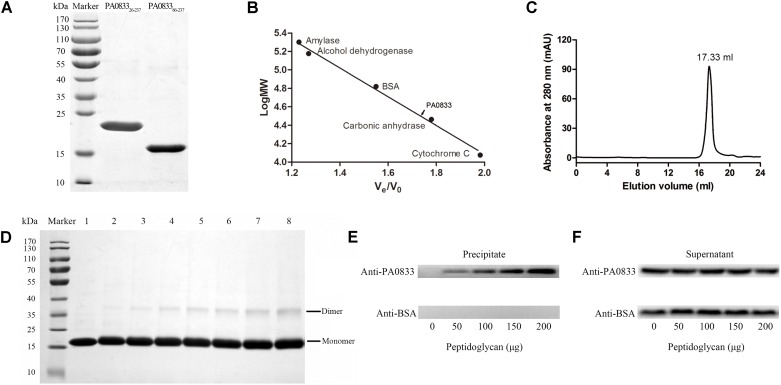
Preparation and characterization of PA0833. **(A)** PA0833_26-237_ and PA0833_86-237_ were purified and analyzed by SDS–PAGE. **(B,C)** Gel-filtration analysis of PA0833_86-237_. The standard curve was built by protein standards (amylase, alcohol dehydrogenase, BSA, carbonic anhydrase, and cytochrome **(C)**. The elution volume of PA0833_86-237_ was 17.33 ml. **(D)** SDS–PAGE analysis of PA0833_86-237_ after treatment with different concentrations of glutaraldehyde. Lane 1 shows the native PA0833_86-237_. Lanes 2–8 show the formation of dimers or aggregates with an increasing concentration of glutaraldehyde (0.01, 0.05, 0.1, 0.2, 0.3, 0.4, and 0.5%). **(E,F)** BSA was used as a negative control. **(E)** Detection of PA0833_86-237_ and BSA from the insoluble precipitates by western blot. **(F)** Detection of free proteins in the supernatant.

As shown in **Figure 3A**, PA0833_26-237_ and PA0833_86-237_ expressed in *E. coli* were soluble, and the purity was up to 95% as determined by SDS–PAGE after two rounds of chromatography. The molecular weights of the two proteins were in accordance with their predicted molecular masses (22.6 and 16.4 kDa for PA0833_26-237_ and PA0833_86-237_, respectively). The C-terminal domain of OmpA family proteins typically form dimers and are able to bind to peptidoglycan *in vitro*. Therefore, we determined the oligomeric states of PA0833_86-237_ by gel-filtration and cross-linking analyses. As shown in **Figure 3C**, the elution volume for PA0833_86-237_ was 17.33 ml, and the molecular weight of PA0833 was calculated as 33.2 kDa according to the standard curve (**Figure 3B**). The theoretical molecular weight of PA0833_86-237_ is 16.8 kDa, and thus it appears as a dimer in solution. This result was further confirmed by the cross-linking assay, where a band corresponding to dimerized PA0833_86-237_ was detected in the gel in the presence of glutaraldehyde, and the amount of the linked PA0833 dimer increased in a glutaraldehyde dose-dependent manner (**Figure 3D**). As a negative control, BSA did not form cross-links in the presence of glutaraldehyde (Supplementary Figure S2).

We next assessed the binding capacity between soluble purified PA0833_86-237_ to insoluble peptidoglycan. As shown in **Figure 3E**, PA0833_86-237_ was detected in the precipitate fraction, whereas the BSA negative control was not observed. In addition, the density of PA0833_86-237_, which binds to insoluble peptidoglycan, increased when the amount of peptidoglycan increased, suggesting that PA0833_86-237_ binds peptidoglycan in a dose-dependent manner. Furthermore, PA0833_86-237_ was also observed in the supernatant fractions, indicating that PA0833_86-237_ was present in excess in the binding assay (**Figure 3F**).

### PA0833 Facilitates Bacteria Survival in a Stressful Environment

**FIGURE 4:**
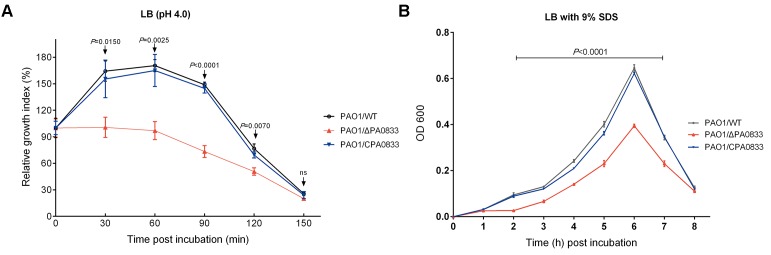
Analysis of the contribution of PA0833 to bacterial resistance to stress. **(A)** Acid resistance of PAO1/ WT, PAO1/ΔPA0833, and PAO1/CPA0833. The three strains of PAO1 were grown in LB (pH 4.0) for 30, 60, 90, 120, and 150 min, respectively. **(B)** The SDS resistance of PAO1/WT, PAO1/ΔPA0833, and PAO1/CPA0833. Bacteria were grown in LB culture that contained 9% SDS for 8 h. The absorbency at 600 nm of the three strains of PAO1 was recorded every hour. The data are shown as the mean ± SD. The differences were compared to determine their statistical significance using one-way ANOVA (ns = no significance). The *P-*values shown in the figure were the statistical difference between PAO1/WT and PAO1/ΔPA0833, and there was no statistical difference between PAO1/WT and PAO1/CPA0833.

To test the function of PA0833 *in vitro*, we first knocked out the PA0833 gene in PAO1 using the suicide vector pCVD442 to generate PAO1/ΔPA0833. And then we constructed complementary plasmid pDN18-PA0833, and electroporated them into the PA0833 mutant strain to generate PAO1/CPA0833, which was confirmed by the results of indirect immunofluorescence (Supplementary Figure S3). No significant difference in the growth rate between PAO1/WT and these two mutants was observed under normal culturing conditions (Supplementary Figure S4). However, in acidic culture medium (pH 4.0), PAO1/WT grew significantly better than PAO1/ΔPA0833, its growth index did not decline until 60 min after incubation (**Figure 4A**). The PAO1/ΔPA0833 strain was sensitive to the acidic environment, as its growth index decreased shortly after incubation. The complement of pDN18-PA0833 in this strain significantly changed the acid resistance ability, which was similar to that of the PAO1/WT, as no significant difference was found between PAO1/CPA0833 and PAO1/WT (**Figure 4A**). This result suggested that PA0833 is partly involved in the acid resistance.

A similar pattern was observed in the presence of the detergent SDS (**Figure 4B**). When the PA0833 mutants were grown in LB with 9% SDS, significant differences were observed from 2 to 7 h after incubation (**Figure 4B**). The PAO1/WT and PAO1/CPA0833 strain grew significantly better than the PAO1/ΔPA0833 mutant, and there was no significant difference in the growth rate between the two strains (**Figure 4B**). This result suggested that PA0833 is responsible for bacterial detergent resistance.

### PA0833 Is Involved in the *P. aeruginosa* Virulence

**FIGURE 5:**
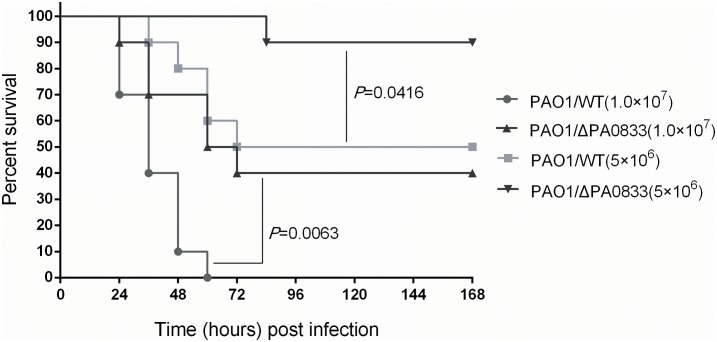
Analysis of the contribution of PA0833 to bacteria virulence. BALB/c mice (*n* = 10) were challenged with PAO1/WT or PAO1/ΔPA0833 at 1.0 × 10^7^ or 5.0 × 10^6^ CFUs/mouse by intratracheal injection, respectively. The survival rate was monitored for 1 week. The *P*-value was calculated using the Mantel–Cox log-rank test.

Since PA0833 provided protective immunity against *P. aeruginosa* infection, we speculated that this protein may be involved in the pathogenicity of the bacterium. With this in mind, we infected mice intratracheally with different doses [LD50 (5 × 10^6^) CFU/mice, 2 × LD50 (1 × 10^7^) CFU/mice] of the PAO1/WT or PAO1/ΔPA0833 strains, and the survival of the mice was monitored for 1 week. As shown in **Figure 5**, at the end of the observation period, mice challenged with the PAO1/ΔPA0833 mutant exhibited higher survival rates (90 and 40%) than the PAO1/WT group (50 and 0%, *P*_LD50_ = 0.0063, *P*_2 × LD50_ = 0.0416, respectively). These data demonstrated that the virulence of PAO1 was decreased in the PA0833 knock out strain, indicating that PA0833 is involved in *P. aeruginosa* virulence.

*Pseudomonas aeruginosa* strains that overproduce alginate, also known as mucoid strains, are associated with chronic endobronchial infections in cystic fibrosis patients (Damron et al., 2012). Through GEO database analyses, we observed that the expression of PA0833 is increased in *P. aeruginosa* strains overexpressing alginate. As shown in Supplementary Figure S5A, in an alginate overproduction mutant strain of PAO1, the expression of PA0833 was significantly higher than that in a PAO1 mutant strain that produces low amounts of alginate [*P* = 0.0163, GEO accession: GSE35248 (Damron et al., 2012)]. Furthermore, the expression of PA0833 in a mucoid strain from a cystic fibrosis (CF) patient was 7.3-fold higher than that in a non-mucoid *P. aeruginosa* strain [Supplementary Figure S5B, GEO accession: GSE9621 (Rao et al., 2008)]. Moreover, the expression of PA0833 in *P. aeruginosa* strains isolated from cystic fibrosis lungs was significantly higher than that in PAO1 [Supplementary Figure S5C, *P* = 0.0147, GEO accession: GSE7704 (Son et al., 2007)]. These results indicated that the expression of PA0833 may be associated with the expression of alginate. The ability of *P. aeruginosa* to produce chronic infection is based in part on its ability to form biofilms, in which alginate is the major polysaccharide. Thus, conversion to the mucoid phenotype in *P. aeruginosa* is associated with a significant increase in morbidity and mortality (Alkawash et al., 2006). These studies further suggested that PA0833 may be participated in the establishment of chronic infection by *P. aeruginosa.*

## Discussion

The OmpA family of outer membrane proteins is characterized as a group of genetically related, surface-exposed porin proteins (Confer and Ayalew, 2013). Typically, these proteins have a high copy number and are highly conserved among different species of Gram-negative bacteria (Confer and Ayalew, 2013). The N-terminal domain of OmpA family proteins typically forms an eight-stranded, anti-parallel β barrel that is embedded in the outer membrane, whereas the C-terminal domain is globular, forms a dimer and is associated with peptidoglycan (Reusch, 2012b). In this study, PA0833 was observed to exist as a dimer in solution and was able to bind to peptidoglycan. The sequence and structure of the C-terminal domain of PA0833 (PA0833_86-237_) was observed to be highly conserved with OmpA family proteins, and two conserved peptidoglycan-interacting residues were also observed in PA0833. In contrast, sequence alignment indicated that the N-terminal domain of PA0833 was not conserved among OmpA family proteins, several deletions were observed in PA0833 when compared with other proteins (Supplementary Figure S1C). Meanwhile, there was no beta barrel domains as predicted by TBBpred, only two trans-membrane domain were predicted by TMHMM, which embedded the protein in the outer membrane of the bacterial. Therefore, we speculated that PA0833 is an OmpA C-like protein.

Because of the high abundance and surface exposure properties of OmpA family proteins in the bacterial outer membrane, these proteins have been reported to participate in a variety of pathogenic pathways and play a key role during bacterial infection (Hancock and Brinkman, 2002; Krishnan and Prasadarao, 2012; Reusch, 2012a). For example, they are involved in bacterial virulence, adhesion and invasion, and in interactions with surface receptors on host cells (Smith et al., 2007; Fito-Boncompte et al., 2011; Sugawara et al., 2012). In addition, these proteins are important for maintaining the integrity of the outer membrane and can stimulate strong antibody responses, with anti-OmpA family proteins antibodies reported to be bactericidal, opsonic, and protective (Zhang et al., 2016; Kazemi Moghaddam et al., 2017; Maccarini et al., 2017; Tang et al., 2017). As a result, OmpA family proteins are promising candidates for vaccine development, and these proteins from a number of bacteria have been reported to be able to induce protective immunity against bacterial infection, some vaccines contain OmpA family proteins even entered phase III clinical trials, such as OprF from *P. aeruginosa* (Price et al., 2001; Oldfield et al., 2008; Peluso et al., 2010; Ayalew et al., 2011; Hounsome et al., 2011; Westritschnig et al., 2014; Zhang et al., 2016; Hassan et al., 2017).

According to our results, PA0833 may function as a novel virulence factor in *P. aeruginosa* for the following reasons. First, PA0833 knockout led to an impaired ability of *P. aeruginosa* cells to resist environment stress, suggesting that PA0833 may be essential for maintaining the integrity of the bacterial cell wall to enhance its adaptability to complex environments and improve the survival of bacteria. Second, the virulence of PAO1 was decreased after PA0833 knockout. Third, the expression of PA0833 was higher in *P. aeruginosa* strains isolated from cystic fibrosis lungs, indicating PA0833 may be associated with the biosynthesis of biofilms and the establishment of chronic infections by *P. aeruginosa*.

As an outer membrane protein, we speculate that PA0833 may interact with the receptors on host cells, such as alveolar epithelial cells and macrophages, to activate substantial signaling pathways to exert its biological effects. We are currently screening for possible receptors of PA0833 in different cell lines and have identified several significantly up-regulated genes in the NOD-like receptor (NLR) signaling pathway, such as NAIP, BIRC3 and HSP90AA1 (Supplementary Figure S6). These results indicate that the Nod-like family of pattern recognition receptors are involved in the innate immune response to *P. aeruginosa*. Further studies will focus on the pathogenic mechanism of PA0833 and the immune response mechanism to PA0833.

Our findings also confirmed that immunization with PA0833 induced strong immune responses and resulted in reduced bacterial loads as well as decreased pathology, inflammatory cytokine expression and inflammatory cell infiltration after *P. aeruginosa* infection. PA0833 was able to induce a protective efficacy against *P. aeruginosa* lethal sepsis and pneumonia murine models. Therefore, as an OmpA C-like protein, PA0833 is a potentially promising vaccine candidate for combating *P. aeruginosa* infection.

In this study, two different truncated forms of PA0833, termed PA0833_26-237_ and PA0833_86-237_, were constructed. The former was used as a vaccine candidate to evaluate the protective efficacy of this protein, primarily because this construct contained the outer membrane domains of this protein, which may be the key domain that induces protective immunity since it is surface exposed. The latter domain was used to evaluate the homology of PA0833 with OmpA proteins because it contains a unique and intact domain that functions similarly to the OmpA family proteins.

## Conclusion

PA0833 is an OmpA C-like protein that was observed to exist as a dimer in solution and was able to bind to peptidoglycan *in vitro*. In addition, PA0833 contributed to bacterial environment stress resistance and virulence. Furthermore, immunization with PA0833 significantly reduced acute systemic infection and pneumonia due to *P. aeruginosa* in mice. Therefore, PA0833 can be regarded as a novel *P. aeruginosa* vaccine candidate. Further studies on PA0833 will focus on its involvement in crosstalk between *P. aeruginosa* and host cells to explain its pathogenic and immune protection mechanisms.

## Author Contributions

JyZ, FL, and QZ designed the research. FY, JG, and JtZ conducted the experiments, analyzed the data, wrote the main manuscript text, and prepared the figures and tables, LL, HJ, and JZ helped to conduct the experiments, HZ and QZ contributed to writing the manuscript and supervised the project. FL and JyZ helped with the discussion of results and manuscript refinement. All authors reviewed the manuscript.

## Conflict of Interest Statement

The authors declare that the research was conducted in the absence of any commercial or financial relationships that could be construed as a potential conflict of interest.

## Footnotes

1 https://www.ncbi.nlm.nih.gov/geoprofiles
