# Supplementary material for: PA0833 Is an OmpA C-Like Protein That Confers Protection Against Pseudomonas aeruginosa Infection
Source: Front Microbiol. 2018 May 23;9:1062. doi: 10.3389/fmicb.2018.01062 (PMC5974059; doi:10.3389/fmicb.2018.01062)
Supplement: Supplementary file 1 [file Table_1.PDF]

Supplementary Table S1. The PCR reaction systems and conditions of amplification the gentamycin resistance (GmR) gene from the plasmid pJQ200SK.

pJQ200SK plasmid (Sangon Biotech, China)	0.5 μl
10 × pfu buffer	5 μl
dNTP (25mM)	0.4 μl
Primers P1 (50pmol/μl)	0.5 μl
Primers P2 (50pmol/μl)	0.5 μl
pfu DNA polymerase (5U/μl, Thermo Scientific, USA)	0.5 μl
dH_2O	42.6 μl
Total	50 μl

Step	Temperature	Time
1	95 degree	5 min
2	95 degree	30 sec
3	55 degree	45 sec
4	72 degree	60 sec
	Repeat 2-4	20 cycles
5	72 degree	7 min

Supplementary Table S2. The PCR reaction systems and conditions of amplification the 5'- and 3'-regions of PA0833.

PAO1	0.5 μl
10 × pfu buffer	5 μl
dNTP (25mM)	0.4 μl
primers-P3/P4 (50pmol/μl)	0.5 μl
Or primers-P5/P6 (50pmol/μl)	0.5 μl
DMSO (5%, Sangon Biotech, China)	2.5 μl
pfu DNA polymerase (5U/μl, Thermo Scientific, USA)	0.5 μl
dH_2O	40.6 μl
Total	50 μl

Step	Temperature	Time
1	95 degree	5 min
2	95 degree	30 sec
3	68 degree	90 sec
	Repeat 2-3	20 cycles
4	72 degree	7 min

Supplementary Table S3. The PCR reaction systems and conditions of fusion PCR.

primers-P1/P2 PCR product	2 µl
primers-P3/P4 PCR product	2 µl
primers-P5/P6 PCR product	2 µl
10×pfu buffer	5 µl
dNTP (25mM)	0.4 µl
primers-P3 (50pmol/µl)	0.5 µl
primers- P6 (50pmol/µl)	0.5 µl
DMSO (5%, Sangon Biotech, China)	2.5 µl
pfu DNA polymerase (5U/µl, Thermo Scientific, USA)	0.5 µl
dH_2O	34.6 µl
Total	50 µl

Step	Temperature	Time
1	95 degree	5 min
2	95 degree	30 sec
3	68 degree	3.5 min
	Repeat 2-3	20 cycles
4	72 degree	7 min
