# Supplementary material for: PA0833 Is an OmpA C-Like Protein That Confers Protection Against Pseudomonas aeruginosa Infection
Source: Front Microbiol. 2018 May 23;9:1062. doi: 10.3389/fmicb.2018.01062 (PMC5974059; doi:10.3389/fmicb.2018.01062)

Supplementary Fig. S1. Bioinformatics prediction of PA0833.

(A) Results output for TMHMM Server 2.0 of PA0833. (B) Crystal structure of the C-terminal domain of OmpA from Acinetobacter baumannii (left) and the putative structure of the C-terminal domain of PA0833 modeled by SWISS-MODEL (right). (C) Sequence alignment of PA0833 with its homologous proteins from different species.

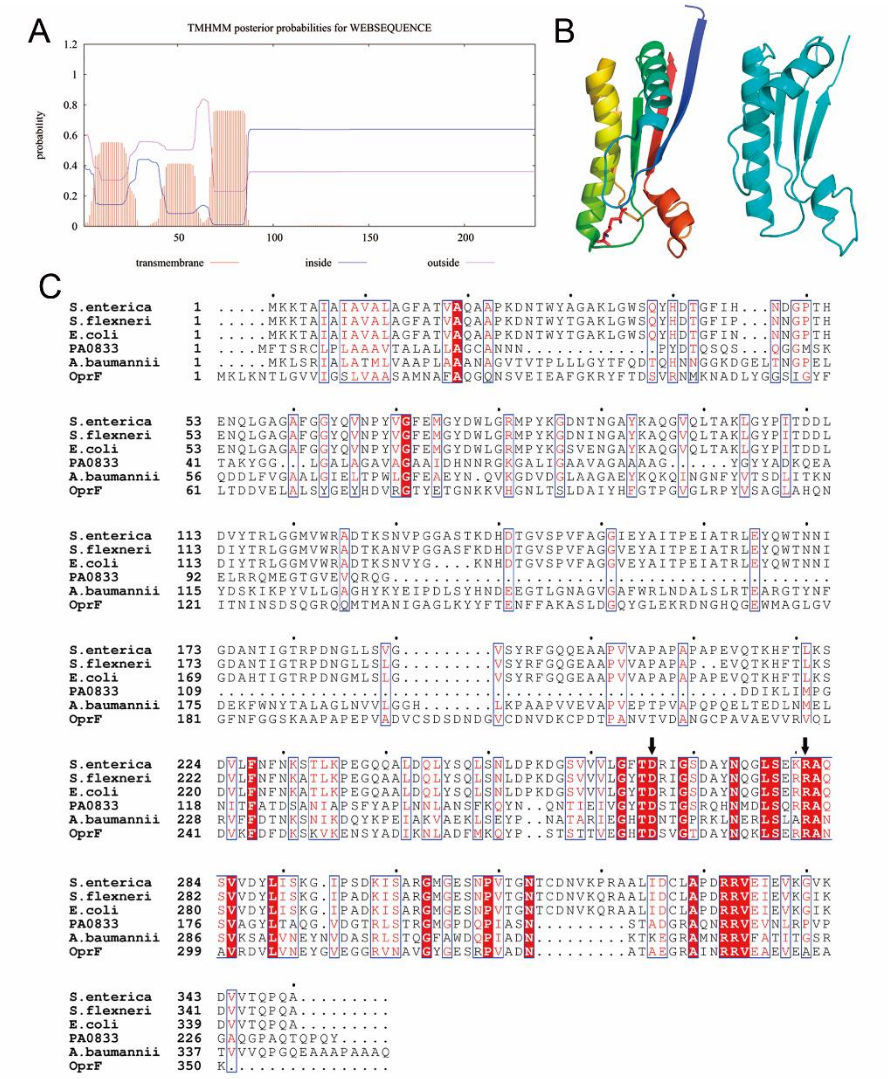

Supplementary Fig. S2. SDS-PAGE analysis of BSA after treatment with different concentrations of glutaraldehyde.

Lane 1 was native BSA. Lanes 2 to 8 were BSA incubated with an increasing concentration of glutaraldehyde (0.01%, 0.05%, 0.1%, 0.2%, 0.3%, 0.4%, and 0.5%).

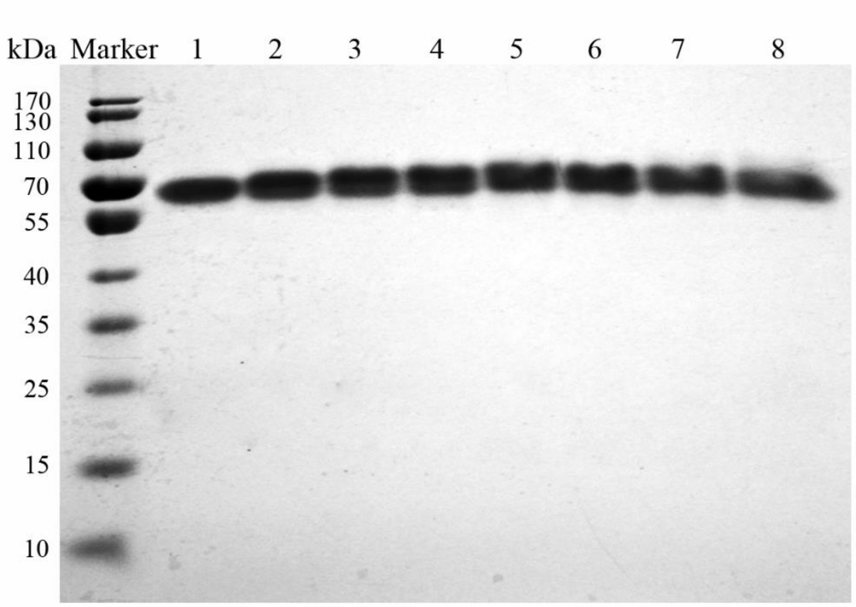

Supplementary Fig. S3. Indirect immunofluorescence analysis of PAO1 isogenic mutants.

Indirect immunofluorescence was conducted to confirm the PAO1 isogenic mutants. (A,C) No immunofluorescence was detected in the absence of anti-PA0833 pcAb or PA0833 gene. (B,D) Positive, indirect immunofluorescence signals indicated binding of anti-PA0833 pcAb with PAO1/WT or PAO1/CPA0833. This study was performed twice, with similar results.

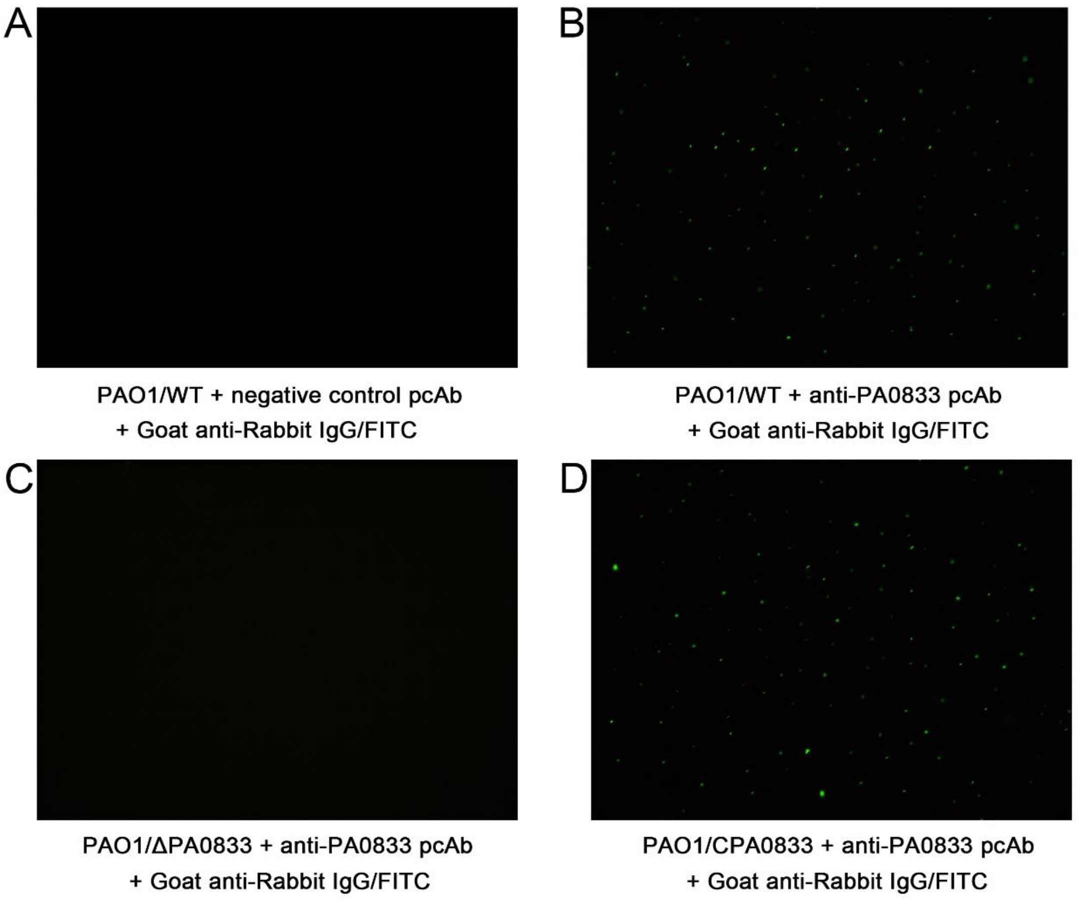

Supplementary Fig. S4. The growth curve of PAO1 isogenic mutants.

Bacteria in the exponential growth phase were harvested and adjusted to 1.0 × 10^9 CFUs/ml in fresh LB. Next, 1/100th of the bacterial suspension was inoculated in fresh LB and incubated for 24 hours at 37℃. Samples were collected for determination of the absorbance at 600 nm every hour. There was no statistical difference between PAO1/WT and these two mutants in the growth rate.

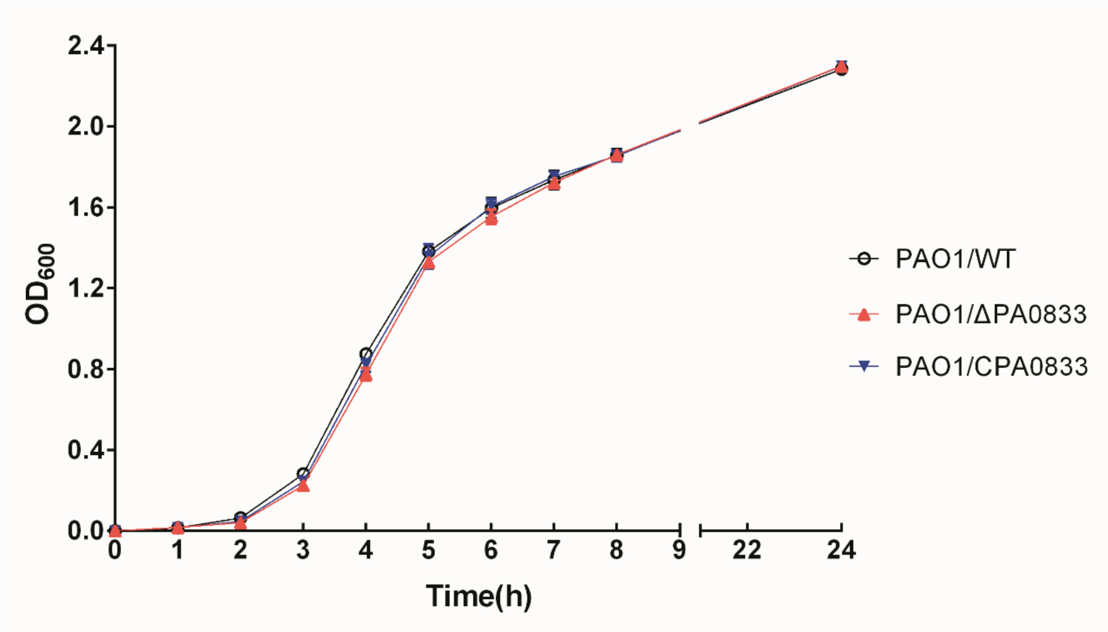

Supplementary Fig. S5. Analysis of the contribution of PA0833 to bacteria virulence. (A) The expression of PA0833 in an alginate overproduction mutant strain and in a low alginate production mutant PAO1 strain. The GEO accession was GSE35248. (B) The expression of PA0833 in a mucoid strain from a cystic fibrosis (CF) patient and a non-mucoid *P. aeruginosa* strain. The GEO accession was GSE96219. (C) The expression of PA0833 in *P. aeruginosa* strains isolated from cystic fibrosis lungs and in PAO1. The GEO accession was GSE7704. The data (A-C) are shown as the means ± SD. The differences were compared to determine their significance using Student's t-test.

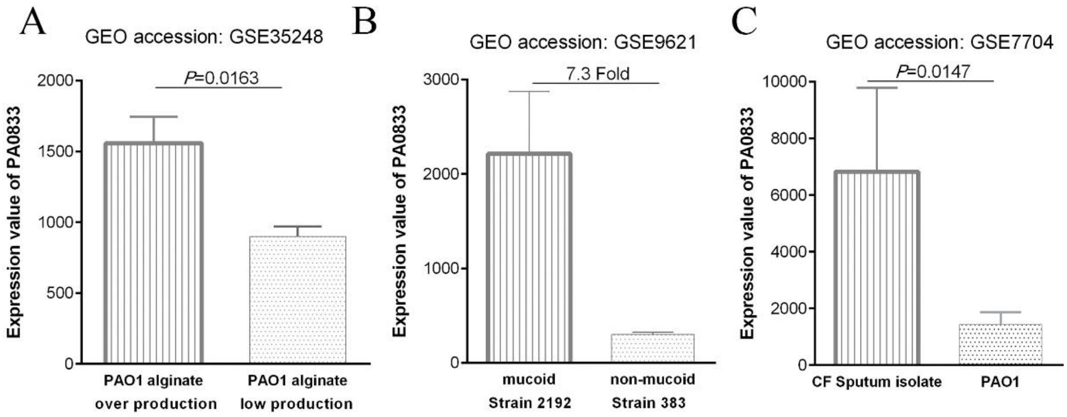

Supplementary Fig. S6. Relative expression of gene NAIP, BIRC3 and HSP90AA1 in A549 cells were detected by qRT-PCR.

PA0833 was added to a final concentration of 10 μg/ml to A549 cells (1x10^6 cells per well in a 6-well plate, in triplicate) and incubated at 37 ℃ in a 5% CO_2 humidified incubator for 24 hours. And then mRNA was extracted from cells and analyzed by qRT-PCR. ACTβ and GAPDH were chosen as housekeeping genes to normalized expression of samples. The data are shown as the mean ± SD. The differences were compared to determine their statistical significance using Student’s t-test.

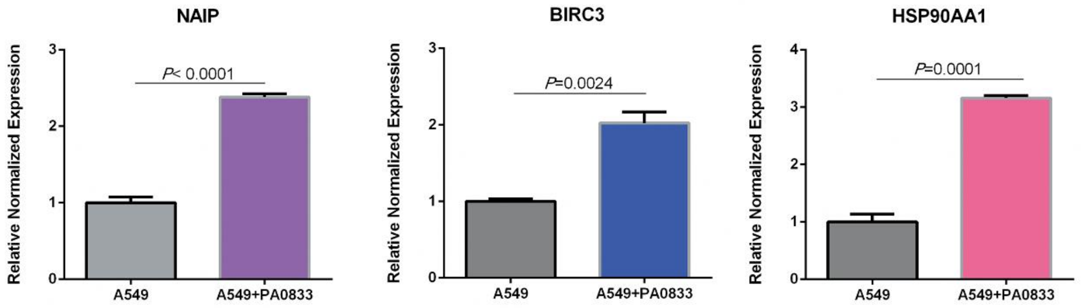

Supplement: Supplementary file 2 [file Image_1.PDF]
